# A case report on the effect of rituximab on pyothorax-associated lymphoma

**DOI:** 10.1097/MD.0000000000018393

**Published:** 2019-12-16

**Authors:** Fei Wang, Hai Lan

**Affiliations:** a College of Integrated Chinese and Western medicine, Gansu University of Chinese Medicine, Lanzhou; b Department of Hematology, First Affiliated Hospital of Guangzhou University of Chinese Medicine, Guangzhou, China.

**Keywords:** nosogenesis, pyothorax-associated lymphoma, rituximab

## Abstract

**Rationale:**

Pyothorax-associated lymphoma (PAL) is a rare type of malignant pleural lymphoma. Most lymphomas are normally discovered around 20 to 50 years after tuberculosis infection. In China, there have been few reports about PAL cases so far. We report a case of a patient, whose tuberculosis and lymphoma were diagnosed concurrently.

**Patient concerns:**

The patient, a 76-year-old male, was reported to our hospital on March 13, 2015. He had recurrent shortness of breath during the previous 2 years of routine activities solely. His symptoms became more serious which was manifested by edema of lower limbs 1 day before his admission to our hospital.

**Diagnoses:**

Doctors reached the diagnosis of PAL based on the patient's pathologic cell morphology and immunohistochemistry. The chest computed tomography examination revealed that there were pleural effusions on both sides, and some extent of compressive atelectasis in the lower parts of the inflamed lungs yet without space-occupying lesions. There were multiple small nodules which may be benign in the right upper lung.

**Interventions:**

The current first-line treatment for diffuse large B-cell lymphoma is the cyclophosphamide, adriamycin, vincristine, prednisone (CHOP) protocol. Given that the patient had cardiac diseases and cardiotoxicity of anthracyclines, doctors decided to adopt rituximab with cyclophosphamide, vincristine, and prednisone chemotherapy without anthracyclines.

**Outcomes:**

The treatment effect was obvious after one cycle of chemotherapy. The patient's pleural and pericardial effusions were significantly reduced. With the chemotherapy protocol above continuously adopted, pleural and pericardial effusions did not increase in multiple reexaminations on October 25, 2015, February 15, 2016, and August 10, 2016.

**Lessons:**

Analytical research revealed that chemotherapy with rituximab can increase the complete remission rate of non-Hodgkin lymphoma, reduce the possibility of failure and relapse, and prolong disease-free and overall survival. Moreover, there is no significant increase in adverse drug reactions compared with the effect of chemotherapy with CHOP alone. In the case of this patient, chemotherapy with rituximab was safe and efficacious.

## 1 Introduction

Pyothorax-associated lymphoma (PAL), a rare kind of malignant pleural lymphoma and closely related to chronic pyothorax (especially caused after tubercle bacillus infection), was first discovered in Japan and now has the highest morbidity rate.^[1]^ There have been reports of PAL in Europe; however, few cases have been reported in China. In 2004, the World Health Organization identified PAL as a separate type of diffuse large B-cell lymphoma (DLBCL). Additionally, by researching PAL cases, Epstein–Barr virus (EBV) infection has an important bearing on the development of the disease. For many patients with PAL, the evidence of EBV infection can be noted.^[1]^ According to literature, most diagnoses of PAL cases were suspicious.^[2]^ The diagnoses are often grounded on 3 clinical features that patients are with a history of tuberculosis, a pathologic type of DLBCL (showed by the pathology of pleural and pericardial effusions), and an evidence of EBV infection. The characteristics of this patient are different from those reported in most cases. PAL is commonly seen in chronic tuberculosis or tuberculous pleurisy. Most lymphomas are discovered about 20 to 50 years after tuberculosis infection, whereas for this patient tuberculosis and lymphoma were diagnosed concurrently. Moreover, in most PAL cases, a biopsy of pleural masses can help confirm lymphoma. However, the patient's imaging did not suggest a pleural nidus. The patient was then examined with pleural and pericardial effusion smears, as well as cell morphology and immunohistochemistry of cell sediment sections.

## 2 Case report

The patient, a 76-year-old male, was admitted to our hospital on March 13, 2015 due to recurrent shortness of breath after activities for over 2 years, which became more serious when it manifested with edema of lower limbs 1 day previously. The patient suffered from: coronary heart disease with unstable angina pectoris, triple-vessel disease, and permanent atrial fibrillation, and an extremely risky 3rd degree high blood pressure. With consideration of the possibility of insufficient cardiac functions, the patient was admitted to the Cardiovascular Department for heart failure treatment. Examinations upon admission revealed shortness of breath, thick breath sounds in both lungs, decrease in breath sounds on both bottom sides of the lungs, small number of dry and wet rales, and no pleural friction sounds. The apical pulse moved slightly to the left; the border of cardiac dullness expanded to the left and right, with a heart rate of 120 beats per minute and arrhythmia. In the aortic valve, 3/6-degree diastolic respiratory murmur can be heard in the left armpit, with no pericardial fricative sound. The related examinations were improved after the patient's admission to our hospital. Myocardial markers, cardiac enzymes, and B-type brain natriuretic peptides did not increase. Temporarily, the symptoms were not considered to be caused by cardiac insufficiency. The chest computed tomography (CT) examination revealed that there were pleural effusions on both sides, and some extent of compressive atelectasis in the 2 lower parts of the inflamed lungs, without space-occupying lesions. There were multiple small nodules which were likely benign in the right upper lung, with massive pericardial effusions. Multiple small lymph nodes existed in the mediastinum. There was aortosclerosis and coronary arteriosclerosis. Coronary heart disease has been considered based on the symptoms while the possibility of cardiac insufficiency remained to be removed. The color Doppler ultrasound examination showed a large number of pleural and pericardial effusions on both sides. The thoracentesis of 2 sides and pericardiocentesis were conducted to relieve compressions of the heart and lungs.

This patient had a large number of pleural and pericardial effusions with unknown causes. Drainage fluid samples of the thoracic and pericardial cavities were tested severally. With hydrothorax T-SPOT (+), combined with patient's serum T-SPOT (+), there was a great possibility of mycobacterium tuberculosis infection. Abnormal lymphocytes could be found in multiple pathologic examinations of pleural fluid. Therefore, the possibility of a lymphoma was hard to rule out. Through pathology consultations, other experts opined that quite a few abnormal lymphocytes, which were centroblastic and had very visible nuclear fissions, existed in both smears of pleural and pericardial effusions, and cell sediment sections. The immunohistochemistry revealed that LCA and CD20 were positive, whereas CK, CR, CD68, CD3, CD43, CD30, and MPO were negative. With paraffin-embedded cell sections added, the immunohistochemistry manifested that CD20 and EBER in situ hybridization were positive, while CD10, Bcl-6, CD138, and Muml were negative. Morphology and immunohistochemistry of heterogenous cells were consistent with those of large B-cell lymphoid tumors. Thus, based on clinical experience and the positive result of pleural T-SPOT tests, the DLBCL associated with chronic inflammation was considered. To further assess the patient's condition, positron emission tomography (PET)/CT examinations were performed:

1. With more spring-strip-shaped lymphomas at a high metabolism rate below the pericardium, combined with pathology, lymphoma infiltration was likely.
2. There was general cardiac enlargement and wall thickening, pericardial effusions, calcification in multiple coronary arteries, as well as common aortic calcification in the whole body.
3. With pleural effusions on both sides and enlarged lymph nodes in mediastina (4.5 groups), the metabolism was slightly increased.
4. Inflammatory lymph node hyperplasia happened on both sides of the neck, in axillary regions, and other mediastina.
5. There were nodules in the right upper lobe with no increase in metabolism, which led to a higher consideration of inflamed nodules. On the grounds of the patient's pathology and PET/CT results, the diagnosis supported the hypothesis of PAL.

Hence, 0.3 g qd isoniazid, 1 g qd pyrazinamide, 0.75 g qd ethambutol hydrochloride, and 0.3 g qd rifampicin were used for anti-tuberculosis therapy. In addition, the rituximab with cyclophosphamide, vincristine, and prednisone (R-CVP) chemotherapy regimen was conducted in five rounds. The specific medications were rituximab (500 mg d0), CTX (0.8 g d1), VCR (2 mg d1), and Pred (50 mg d1–5). According to the Response Evaluation Criteria in Solid Tumors (V1.1), complete remission (CR): normal bone marrow phase or histologic examination, complete disappearance of lymph nodes without contact, CT scan of the lesion <1 mm; partial remission (PR): lesion reduction 50%; Stable (SD): lesion reduced by 25% to 49%; progression (PD): reduction of single or several lesions by 24% or occurrence of new lesions. Total effective rate = (CR + PR)/total cases × 100%. Currently a 5-point scale is applied to both clinical trials including interim analysis and end-of-treatment assessment (Table 1 ). During the entire period of treatment, tests including assessment of extra-nodal sites, which initially showed abnormal results were repeated. After treatment, the patient's condition was stable with no adverse reactions. Re-examinations on October 25, 2015, February 15, 2016, and August 10, 2016 revealed no increase in pleural and pericardial effusion.

**Table 1:**
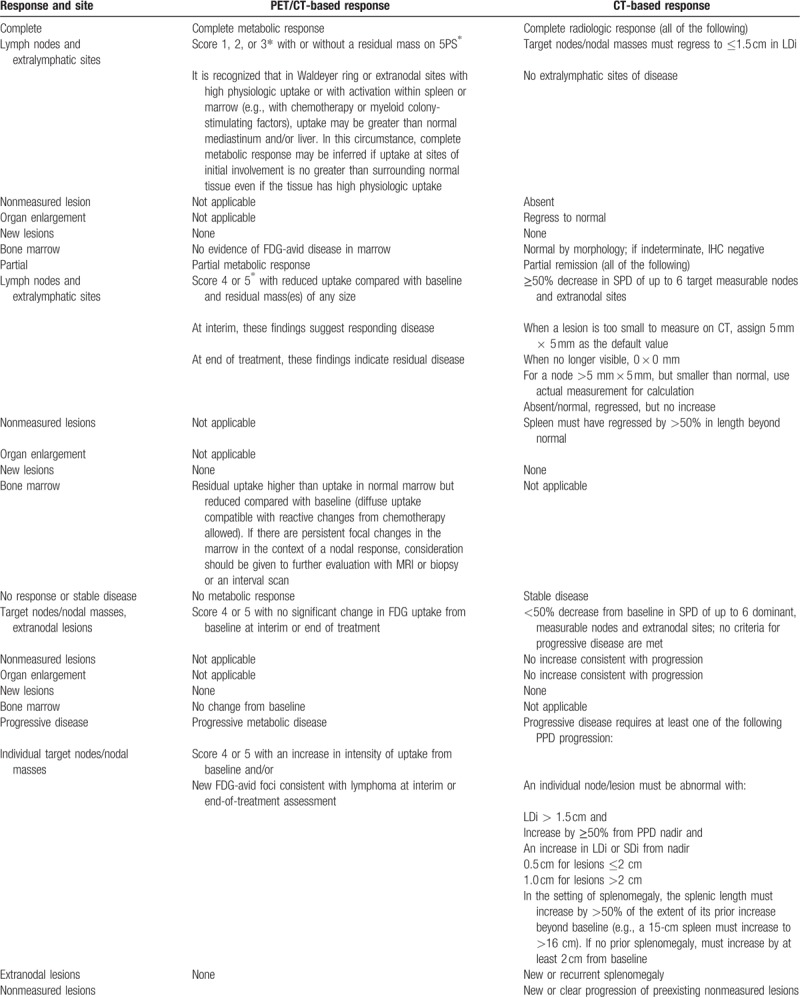
Revised criteria for response assessment.

**Table 1 (Continued):**
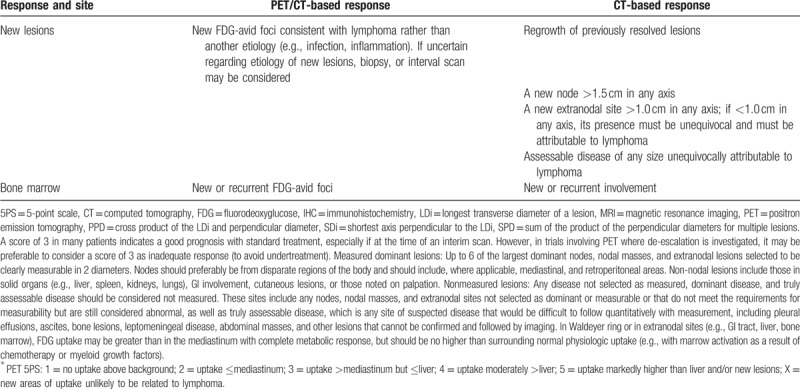
Revised criteria for response assessment.

## 3 Discussion

### 3.1 Basic features of PAL

Through conclusive review of relevant literature, PAL has 3 basic features.^[3,4]^ Patients usually have a history of tuberculosis or tuberculous pleurisy prior to PAL development. Now, it is estimated that chronic inflammatory stimulation leads to abnormal secretion of IL-6 and IL-10, as well as malignant proliferation of large B cells infected with EBV. IL-6 and IL-10 help these cells to evade the immune system's surveillance, thereby causing malignant proliferation of lymphoma cells. IL-6 can accelerate the growth of lymphocytes which express IL-10 and are infected with EBV. IL-6 can also boost the occurrence and development of lymphoma.^[5]^ Pleural masses, mostly located in the pleura and visible around the pyothorax, can be detected by imaging. They may also exist in the lungs adjacent to the pleura and show infiltrative growth, with or without regional lymph node enlargement. The main pathologic type of nidus was diffuse large B-cell non-Hodgkin lymphoma. The immunocytochemistry of tumor cells was characterized by many CD20 and/or MB-1-positive tests, whereas CD3 and CD45RO were not expressed. EBV infection: in most cases, with methods of immunohistochemistry, polymerase chain reaction, and in situ hybridization, the evidence of EBV infection, such as latent membrane protein-1 (LMP-1), EBV-associated nuclear antigen-2 (EBNA-2), and EBV receptor-1 (EBER-1), can be detected among tumor cells.^[1]^ Studies reveal that latently infected cell expressions of EBV and related gene products of EBV, such as EBNA-2, LMP-1, and EBER-1 cause EBV-infected cells to escape the body's immune surveillance without being cleared out by cytotoxic T cells. EBV can cause long-term survival of infected B lymphocytes, thereby inducing the occurrence of lymphoma. In addition, polyclonal B-cell proliferation disorder and malignant lymphocyte monoclonal proliferation can be induced.^[5]^ For organisms with immunodeficiencies, cells expressing EBNA-2 and LMP-1 cannot be eliminated, which may lead to development of lymphoma. Moreover, certain studies have revealed that for patients with PAL, there are special p53 mutations at dipyrimidine sites which may be relevant to long-term exposure to radiation. This may be as a result of artificial pneumothorax surgery and drug use that cause p53 gene mutations.^[6]^

Literature suggest that most diagnoses of PAL cases were suspicious.^[7]^ The diagnoses are often grounded on 3 clinical features that patients are with a history of tuberculosis, a pathologic type of DLBCL, and evidence of EBV infection. These cases are evidenced by tuberculosis infection, DLBCL showed by the pathology of pleural and pericardial effusions, and EBV infection, which support the diagnosis of PAL. The characteristics of this patient are different from those reported in most cases. PAL is commonly seen in chronic tuberculosis or tuberculous pleurisy. Most lymphomas are discovered about 20 to 50 years after tuberculosis infection, whereas for this patient, tuberculosis and lymphoma were diagnosed concurrently. Moreover, in most PAL cases, a biopsy of pleural masses helps in confirmation of lymphoma. However, the patient's imaging did not suggest a pleural nidus. Then the patient was checked with pleural and pericardial effusion smears as well as cell morphology and immunohistochemistry of cell sediment sections. As mentioned above, PAL is a rare disease. The basic features of the disease have been concluded by generations of doctors and scholars based on research on cases available, which, to some extent, have been supported by evidence in follow-up studies. However, it does not indicate that these 3 clinical features are the only diagnostic criteria for PAL, to which cases must conform. There have been reports of cases which are not completely consistent with the characteristics mentioned before.^[7]^ As more and more cases are analyzed and diagnosed, more clinical features of PAL will be discovered.

### 3.2 Identification and diagnosis

The PAL needs to be differentiated from primary exudative lymphoma (PEL). PEL is a subtype of B-cell lymphoma, few cases of which are currently reported in China.^[2]^ PEL has the following characteristics:

1. It usually shows body cavity effusions that exude without tumor masses, and occurs in the chest, abdominal and pericardial cavities, but often involves in only 1 body cavity.
2. It is related to human herpesvirus 8 (HHV8) infection.
3. For most immunodeficient patients, PAL occurs during disease development or in an immunosuppressed state (such as the period after organ transplantation).
4. The PEL cells are polymorphous. Their shapes can take form of immunoblastic cells to plasmablastic ones. They have atypical and eosinophilic cytoplasmic content with a large, round, or irregular nucleus. The immunophenotype CD45 is positive; CD30, CD38, and CD138 are often positive; CD3 is sometimes positive. However, CD19, CD20, and CD79a are usually negative. When HHV8 is detected by in situ hybridization, positive signals often appear in the nucleus. In combination with patient pathologic cell morphology and immunohistochemistry, doctors diagnosed this patient with PAL.

Finally, pyothorax should be distinguished from primary cardiac lymphoma. Primary cardiac lymphoma is a solid tumor, an extranodal lymphoma that invades only the heart and/or the pericardium, accounting for only 0.5% or less of the extranodal lymphoma.^[8]^ The tumor can invade any part of the heart, which is most commonly seen in the right atrium. The tumor is mostly nodular or globular and grows inward. It may also have infiltrative growth in the myocardium, forming nodular or irregular masses, harming the pericardium, and causing pericardial effusions. Quite few violations of other parts occur. In respect of pathologic types, DLBCL is more commonplace, while a small number are T-cell types. The patient's PET/CT scan showed infringement of the pericardium, but no solid tumors were found in the heart and no out-of-cardiac invasion occurred. Thus, the speculation of primary cardiac lymphoma was removed.

### 3.3 Treatment

Currently, no treatment options for PAL have been universally acknowledged. The pathologic type of PAL is DLBCL. And the main treatment is chemotherapy, which is now chiefly combined with rituximab. Analytical studies have demonstrated that the total effectiveness of rituximab in the treatment of NHL has reached 81%.^[9]^ Rituximab is a human-mouse chimeric monoclonal antibody against CD20 produced by gene recombination technology.^[10]^ Since the FDA approved its entrance into market in 1997, it has shown considerable advantages in the treatment of B-cell lymphoma. More than 95% of B-cell lymphoma express the CD20 antigen. The functioning mechanism of rituximab involves killing CD20^+^ B lymphocytes by antibody-dependent and complement-mediated cytotoxicity, inducing apoptosis of B-lymphocyte-associated malignant tumor cells, enhancing the sensitivity of drug-resistant cell lines to chemotherapeutic drugs, and downregulating bcl-2 gene expression.^[11]^ Analytical research reveals that chemotherapy combined with rituximab can increase the CR rate of NHL, reduce the possibility of failure and relapse, and prolong disease-free and overall survival. In comparison with chemotherapy alone, it also results in no significant increase in adverse drug reactions.^[12]^ At the same time, with the patient's serum and pleural effusion T-SPOT (+) considered, the chance of mycobacterium tuberculosis infection and activities could not be underestimated. Chemotherapy can cause dissemination of tuberculosis infection as well as aggravate the condition. But if to deliver exclusively an antituberculosis therapy without containing chemotherapy, lymphoma will continue to develop and lead to worsening. Consequently, on the basis of antituberculosis therapy, patients received chemotherapy combined with rituximab. The current first-line treatment for DLBCL is the cyclophosphamide, adriamycin, vincristine, prednisone protocol. Given that these patients usually have heart-based diseases and the cardiotoxicity of anthracyclines, doctors decided to adopt R-CVP chemotherapy without anthracyclines. The treatment effect was obvious after one chemotherapy cycle. The patient's pleural effusions and pericardial effusions were significantly reduced. With the chemotherapy protocol continuously adopted, reexaminations during the 3 years that followed showed no increase in pleural and pericardial effusion. In the case of this patient, chemotherapy combined with rituximab toward for treatment of PAL was safe and efficacious.

## Author contributions

**Conceptualization:** Lan Hai.

**Data curation:** Wang Fei.

**Formal analysis:** Wang Fei.

**Project administration:** Lan Hai.

**Writing – original draft:** Wang Fei.

**Writing – review & editing:** Wang Fei, Lan Hai.
